# Sequential recovery of alginate from fucoidan extraction by-products of *Nizamuddinia zanardinii* seaweed using green extraction methods

**DOI:** 10.1016/j.ultsonch.2025.107343

**Published:** 2025-04-08

**Authors:** Mehdi Alboofetileh, Samira Jeddi, Mehdi Abdollahi

**Affiliations:** a Persian Gulf and Oman Sea Ecological Research Center, Iranian Fisheries Science Research Institute (IFSRI), Agricultural Research, Education and Extension Organization (AREEO), Bandar Abbas, Iran; b Department of Life Sciences-Food and Nutrition Science, Chalmers University of Technology, Gothenburg, Sweden

**Keywords:** Seaweed, Brown Algae, Fucoidan, Alginate, Ultrasound, Microwave, Biorefinery

## Abstract

The effects of green technologies-ultrasound, microwave and their combined application-on alginate extraction from fucoidan production by-products of brown seaweed (*Nizamuddinia zanardinii*) were compared with the conventional alkaline method. The impact of the extraction methods on the Fourier-transform infrared spectroscopy (FT-IR) spectra, molecular weight, antioxidant, rheological, emulsifying and foaming properties of the recovered alginates was also evaluated. The highest (15.36 % w/w) and lowest (11.88 % w/w) alginate yields were obtained using the microwave and conventional methods, respectively. Using ultrasound (2362 kDa) and ultrasound-microwave (2608 kDa) led to a significant reduction (*p* < 0.05) in the average molecular weight of alginate in comparison to the microwave (3015 kDa) and alkaline methods (3021 kDa). The microwave-extracted alginate showed the highest DPPH (2,2-diphenyl-1-picrylhydrazyl) radical scavenging activity (19.98 %-35.60 %) and ferric reducing antioxidant power (FRAP) (0.138–177 abs) of the extracted alginates. The rheological properties of the alginates were affected by the extraction method, resulting in the highest viscosity in the microwave- and conventionally-extracted alginate. Also, all the extracted alginates showed moderate emulsifying and foaming properties. Overall, the findings highlight the great potential of green technologies to enhance the recovery and functionality of alginate from fucoidan extraction by-products, advancing the efficient biorefining of brown seaweed.

## 1 Introduction

Alginates are derivatives of alginic acids found in the cell wall of brown seaweeds and also some of them can be obtained from some bacteria as capsular polysaccharides [1]. Structurally, alginates consist of β-D-mannuronic acids (M) and α-L-guluronic acids (G) with different arrangements, including homo-polymeric (MM and GG) or hetero-polymeric (MG). Various factors such as seaweed type, harvest season, geographical location, climatic factors, and extraction method/conditions can affect the arrangements of M and G in the structure of alginates [2]. These variations finally lead to the alginates with different properties and functionality [3]. For example, G-rich alginates generally form hard and brittle gels whereas M−rich samples produce more soft and elastic gels [4].

Alginate has shown various bioactivities including antioxidant [5], anti-inflammatory [6], antitumoral [7], immune modulatory [8], inhibition of α‑amylase [9], prebiotic [3], *etc*. Furthermore, alginate possesses appropriate emulsifying and rheological properties as well as gelling, thickening and stabilization of dispersions [5], [10]. Based on these properties and also the purity and structural features, such as M and G distribution pattern, molecular weight, and composition, there are various types and grades of alginates on the market. Accordingly, the price and usage of alginates vary from one type to another. The existence of different types of alginates provides a wide range of biological and functional properties that determine their use in food, cosmetic, pharmaceutical, biomedical, agriculture, textile and paper industries and also their commercial value. In general, the low-grade alginates are used in textiles and printing, while alginates with strong gel-forming capability, high viscosity and near-absence of color, which are more favorable, are employed in the food and beverage industries and also in drug delivery systems and cell immobilization applications. The use of alginate in different products could reduce production costs and improve the economics of the industry [11], [12].

Alginate properties are affected by different parameters, especially seaweed species and extraction methods. *Ascophyllum nodosum, Laminaria hyperborea, Laminaria japonica, Laminaria digitata* and *Macrocystis pyrifera* are the main species used commercially for alginate extraction [11] but there is great interest in exploring other brown seaweeds for their alginate content and its properties to find outperforming or local sources.

The extraction method could affect the extraction yield of alginate and its structure and quality. Alkaline is a conventional method used for the isolation of alginates from algal resources, which is the most investigated method too [5]. Besides the conventional methods, different green or eco-friendly methods such as ultrasound, microwave, supercritical fluids, *etc.,* have been increasingly developed to improve the isolation processes of natural products. For example, several studies have reported alginates extraction from different brown seaweeds using individual enzyme [6], [8], [13], ultrasound [7], [14], microwave [15], [16], [17], [18], hydrostatic pressure-assisted extraction [19] and sequential combination of microwaves and ultrasounds [20].

In our previous studies, we demonstrated a great potential for fucoidan extraction from *N. zanardinii* using conventional and different green techniques [21], [22]. However, after fucoidan extraction, a significant amount of solid fraction remains which is typically discarded. This remaining solid part could be used as a raw material for the extraction of other polysaccharides through a biorefinery process. Herein, a few studies have reported the potential for the alginate recovery from post-fucoidan extraction by-products. For example, Abraham et al. [23] extracted the fucoidan, laminarin and alginate polysaccharides from *Durvillaea potatorum* using a biorefinery process under mechanical stirring. Ummat et al. [24], [25] extracted alginate from the fucoidan extraction by-products of *Fucus vesiculosus* and *A. nodosum* using ultrasound. However, a side-by-side investigation of different green extraction methods and their impact on the recovery and properties of alginate obtained from fucoidan by-products remains unknown to the best of our knowledge.

Therefore, this study aimed to investigate the possibility of improving the alginate extraction efficiency from the fucoidan extraction by-products of *Nizamuddinia zanardinii* using different green technologies including ultrasound, microwave and sequential combination of ultrasound and microwave methods and to compare their effect with the conventional method. The effect of the extraction methods on the Fourier-transform infrared spectroscopy (FT-IR) spectra, molecular weight, antioxidant, rheological, emulsifying and foaming properties of the recovered alginate was also evaluated.

## 2 Material and methods

### 2.1 Chemicals

All chemicals and reagents used in this work were of analytical grade. 2,2-diphenylpicrylhydrazyl (DPPH), Hydrochloric acid (HCl), Potassium bromide (KBr), Sodium carbonate (Na_2_CO_3_)_,_ potassium ferricyanide, Ferric Chloride (FeCl_3_), Trichloro acetic acid (TCA), Carboxymethyl cellulose (CMC) and ascorbic acid were purchased from Sigma-Aldrich and Merck companies. Ethanol and acetone were purchased from Taghtir Khorasan (P.S.G) and Chem-Lab companies.

### 2.2 Algal materials

**Fig. 1:**
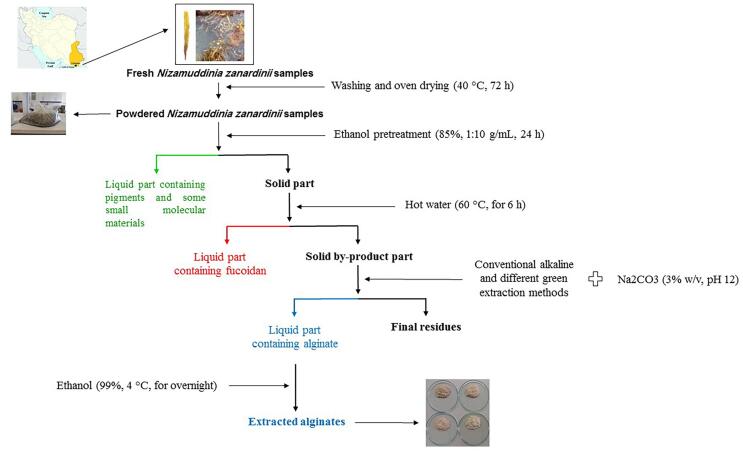
Steps involved in alginate extraction from fucoidan extraction by-products of *N. zanardinii* samples using different extraction methods.

The marine brown algae *N. zanardinii* was freshly collected from the coastal region of the Chabahar beaches in the Province of Sistan and Baluchistan, Iran. Fresh seaweed samples were washed, oven dried (40 °C, 72 h), finely ground into homogeneous powder, sieved through a < 0.5 mm mesh, and stored in a laboratory freezer (−20 °C) until use. Fig. 1 shows the steps involved in alginate extraction from fucoidan extraction by-products of *N. zanardinii* samples using the different extraction methods.

### 2.3 Pretreatments of algal materials for fucoidan extraction

The powdered *N. zanardinii* samples were pretreated with ethanol (85 %, 1:10 g/mL) with constant mechanical stirring (1200 rpm) for 24 h at room temperature (22 ± 2 °C). During this period, the solvent was changed three times to remove pigments and some low molecular weight components. The solid residue was separated by centrifugation (9000 rpm, 10 min) and washed with acetone and dried overnight at room temperature for under a laminar hood. The dried *N. zanardinii* samples were treated by hot water (60 °C, for 6 h) with constant mechanical stirring (1200 rpm). Then, the liquid part was separated for fucoidan extraction and the remaining solid part was collected for the recovery of alginate using the following methods.

### 2.4 Alginate extraction from the fucoidan extraction by-product

#### 2.4.1 The conventional alkaline method

The conventional alkaline extraction of alginate was performed according to Torres et al*.* [1] with some modification. Ten grams of the solid by-product after the fucoidan extraction was placed into glass beakers containing 200 mL of Na_2_CO_3_ solution (3 % w/v, pH 12) and stirred (1200 rpm) for 6 h at 65 °C. After the treatment, the liquid part was separated from the solids by filtration (Whatman filter paper, grade 42) and centrifugation (9000 rpm for 10 min). The collected extracts were mixed with three volumes of cold ethanol (99 %, 2 °C) and the mixture was incubated overnight at 4 °C. The formed alginate fibers were collected and homogenized using a food blender (Model 310P, Pars Khazar Co., Iran). The homogenized alginate was recovered by centrifugation (9000 rpm, 10 min) and washed three times with ethanol (99 %), followed twice by acetone. The recovered alginate was dried at room temperature (22 ± 2 °C) and stored at a freezer (–18 °C) until analyzed. The alginate yield was calculated based on the dried fucoidan extraction by-products.

#### 2.4.2 Ultrasound-assisted method

Ten g of the fucoidan extraction by-product was added to Na_2_CO_3_ solution (3 % w/v, pH 12) and the suspension treated with a high-power ultrasonic probe system (Ultrasonic Technology Development Co., Iran) with a flat tip titanium horn which operated at a frequency of 20 kHz and the power of 200 W. The probe was directly submerged into the suspension and the ultrasonication was performed at 55 ± 2 °C for 15 min (two times). Choosing these extraction conditions was based on our previous studies regarding the polysaccharides extraction by ultrasound at different power, temperature and time, which showed that these conditions resulted in appropriate extraction yield and efficiency. During the extraction, the temperature was controlled using a water bath. After the ultrasound treatment, the liquid fraction was recovered and mixed with 3 volumes of cold ethanol (99 %, 2 °C) and maintained overnight at 4 °C for precipitation. The washing and drying procedures were performed in the same manner as described for the conventional method.

#### 2.4.3 Microwave-assisted method

Ten g of the fucoidan extraction by-product was added to Na_2_CO_3_ solution (3 % w/v, pH 12) and the suspension treated with a microwave apparatus (Model ME341, Samsung CO., Malaysia). Microwave irradiation (700 W) was performed at 90 °C for 15 min (two times). These extraction conditions were chosen based on our previous studies of polysaccharides extraction by microwave at different power, temperature and time, which showed that these conditions resulted in appropriate extraction yield and efficiency. After irradiation, the liquid part was recovered and mixed with 3 volumes of cold ethanol (99 %, 2 °C) and maintained overnight at 4 °C for alginate precipitating. The alginate fibers were homogenized, recovered, washed with ethanol and acetone, and dried as explained for the conventional method.

#### 2.4.4 Sequential combination of ultrasound and microwave-assisted method

Ten g of the fucoidan extraction by-product was mixed with Na_2_CO_3_ solution (3 % w/v, pH 12) and the suspension was first treated by ultrasound at 55 ± 2 °C for 15 min. The supernatant was recovered and the precipitate was extracted using the microwave (90 °C for 15 min) and the supernatant was recovered. Then, the supernatants were combined and mixed with 3 volumes of cold ethanol (99 %, 2 °C) and maintained overnight at 4 °C for alginate precipitation. The remaining steps were performed in the same manner as the conventional method.

### 2.5 Fourier transform infrared (FT-IR) spectroscopy

FT-IR patterns of the alginates were recorded with a FTIR spectrophotometer (Bruker Instruments, Billerica, MA, USA). The samples were mixed with KBr powder, pressed into a disk, and scanned at room temperature in a frequency range of 400–4000 cm^−1^ at a resolution of 4 cm^−1^.

### 2.6 Molecular weight analysis

The molecular weight of the extracted alginates was determined using a high performance size exclusion chromatography column linked to a UV, multi-angle laser light scattering and refractive index detector system (HPSEC–UV–MALLS–RI) according to the method reported by Jeddi et al. [26]. Briefly, 4 mg of each alginate was dissolved in 2 mL of distilled water and heated in a microwave oven (RE-552 W, Samsung, Seoul, South Korea) for 30 s and then filtered through a cellulose acetate membrane (3.0 m pore size; Whatman International) before injection into the HPSEC–UV–MALLS–RI system.

### 2.7 Antioxidant activity measurement

#### 2.7.1 DPPH scavenging activity

Different concentrations (0.0625–0.5 mg/mL) of the extracted alginates were prepared in distilled water. Followed by mixing 100 μL of the alginate solution with 100 μL of the DPPH solution (1 mg DPPH in 25 ml ethanol). Then, the reaction mixture was incubated for 30 min in the dark at room temperature (22 ± 2 °C). Finally, the absorbance of the sample solutions was measured at 515 nm using ELISA microplate reader [8]. The positive control was ascorbic acid (100 µg/mL). The following equation was used for calculating the DPPH radical scavenging activity:

(1) $$DPPH\;scavenging\;activity\;\left(\%\right)\;=\;\frac{Ac-As}{Ac}\times \;100$$

Ac: the absorbance of control (100 μL of ethanol with 100 μL of the DPPH solution)

As: the absorbance of polysaccharide sample solution

#### 2.7.2 Reducing power

First, 200 μL of each alginate solution (0.0625–0.5 mg/mL) was mixed with 500 μL of potassium ferricyanide (1 %) and 500 μL of phosphate buffer (0.2 M, pH 6.6) in 2 mL micro tubes. After incubation at 52 °C for 30 min in water bath, 500 μL of 10 % TCA was added to the tubes and the mixture was centrifuged (10000 rpm, 10 min). After centrifugation, 500 μL of supernatant transferred into new micro tube. Next, 500 μL of distilled water and 100 μL of ferric chloride (Fe^3+^, 0.1 %) were added to the tubes and then, the reaction mixture was incubated for 10 min at room temperature (22 ± 2 °C). Finally, the absorbance of the solutions was measured at 700 nm using ELISA microplate reader [8]. The positive control was ascorbic acid (100 µg/mL).

### 2.8 Rheological properties measurement

Determination of steady-shear flow measurements of alginate solutions was conducted according to the method reported by Flórez-Fernández et al. [7]. Alginate solutions were prepared by dissolving the alginates (2 % w/v) in distilled water under stirring (100 rpm) for 1 h. Then, apparent viscosity of each solution was recorded at a shear rate in the range of 0.01 to 1000 s^−1^ using a dynamic rheometer (Paar Physica, Rheometer MCR 300, Anton Paar GmbH, Austria) at 25 °C under rotation mode using a concentric probe.

### 2.9 Emulsifying properties

The method reported by Fawzy et al. [5] was used for determination of emulsification index (E24) of the extracted alginates. The used positive control was carboxymethyl cellulose (CMC). An aqueous solution of alginate (0.5 % w/v) was mixed separately with sunflower, corn and canola oils (3:2 v/v ratio) and vortexed for 2 min at 2000  rpm. After 24  h, the emulsification index (E24) was determined using the following equation:

(2) $$E24\;\left(\%\right)\;=\;\frac{He}{Ht}\;\times \;100$$

He: the height of the emulsion layer

Ht: the total height of the mixture.

### 2.10 Foaming properties

Foaming properties including foam capacity (FC) and foam stability (FS) of extracted alginate were determined according to the method of Kazemi et al. [27]. Five mL of an alginate solution (0.5 % w/v) was placed in a tube and vortexed (2000 rpm) for 3 min at room temperature (22 ± 2 °C). FC and FS were calculated using the following equations:

(3) $$FC=\frac{the\;total\;\;volume\;\;after\;\;whipping-the\;\;volume\;\;before\;\;whipping}{the\;\;volume\;\;before\;\;whipping}\times 100$$

(4) $$FS=\frac{the\;\;total\;\;volume\;\;after\;\;leaving\;\;at\;\;room\;\;temperature\;\;for\;\;30\;\;min-the\;\;volume\;\;before\;\;whipping}{the\;\;volume\;\;before\;\;whipping}\times 100$$

### 2.11 Statistical analyses

All measurements were done in triplicate, and the data were presented as mean values ± SD. SPSS statistical software (version 16) was used for statistical data analyses. One-way ANOVA and Duncan's test (p < 0.05) were performed to calculate the differences between the concentrations of alginate in different assays.

## 3 Results and discussion

### 3.1 Extraction yield of alginate

**Fig. 2:**
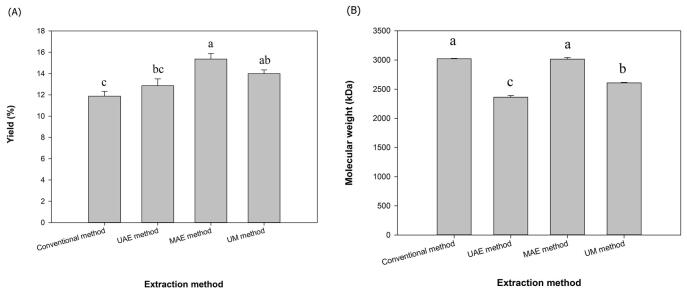
Extraction yield (A) and molecular weight (B) of alginates extracted from fucoidan extraction by-products of *N. zanardinii* samples using different extraction methods (n = 3, means ± SD). The letters a, b, c indicate a significant difference (p < 0.05) between the different extraction methods.

Fig. 2A shows the extraction yield of alginate from fucoidan extraction by-products of *N. zanardinii* samples. The alginate yield varied from 11.88 % to 15.36 % w/w. This extraction yield was higher than the yield of alginate extracted from *Cystoseira myrica* (2.2 %), *Cystoseira trinode* (3.26 %), *Sargassum dentifolium* (3.28 %), and *Sargassum latifolium* (4.3 %), which extracted using 3 % sodium carbonate [28]. Rostami et al [8] reported that the pre-treatment of *C. peregrina* samples by cellulase enzyme (5 %, pH 4.5, 50 °C, 24 h) led to the highest alginate yield (6.6 %) compare to the hot water (65 °C, 3 h, 3 times), acidic (0.1 M Hcl, pH 2, 65 °C, 3 h, 3 times) and alcalase enzyme (5 %, pH 8, 50 °C, 24 h) methods. In another study, conventional alkaline extraction (2 % sodium carbonate at 99 °C for 3 h under stirring) led to the higher alginate yield from *Padina gymnospora* (16 %), *Sargassum vulgare* (17 %), *Padina antillarum* (22 %), *Sargassum natans* (23 %), *M. pyrifera* (26 %) and *L. digitata* (29 %) [4]. Significantly higher alginate yield (56 %-96 %) was obtained from *A. nodosum* using conventional chemical extraction (3 % sodium carbonate, 70 °C, 3 h, 3 times), microwave-assisted extraction (90 °C, 15 min, 3 times), ultrasound-assisted extraction (35 min, 3 times) and a combination of enzyme-assisted (pH 4.5, 50 °C, 24 h) and conventional chemical extraction (3 % sodium carbonate, 70 °C, 3 h, 3 times) [3]. These variations in the alginate extraction yield could have originated from the variation in the parameters e.g. the species type, harvesting seasons, growing locations of the seaweed [8], [29]. The extraction conditions such as temperature, time, ratio of solid to the solvent, alkaline concentration and type of isolation method also have proven to have a determinant effect on the alginate yield [30], [31]. At present work, the microwave-assisted method showed the highest extraction yield (15.36 %) which was significantly higher than the conventional alkaline (11.88 %), ultrasound-assisted extraction (12.865) and combination of ultrasound-microwave (13.99 %) methods. This higher alginate yield could be related to the higher temperature (90 °C) used in the microwave method compared to other methods. Similar results previously reported by Chen et al. [32] for the extraction of polysaccharides from *Semen Cassiae* by different methods. In that study, the microwave method had higher extraction yield in comparison to the ultrasound and heating solvent extraction methods. In another study, Rostami and Gharibzahedi [33] also reported that the polysaccharides yield from jujube by microwave (9.07 %) was higher than conventional (6.72 %) extraction method. However, Yuan and Macquarrie [15] reported that the extraction yield of fucoidan from *A. nodosum* using microwave method (16.08 %) was lower than those obtained using the conventional method (20.98 %). This reduction could be originated from the difference in the extraction time which was significantly shorter in the microwave method (15 min) compared to the conventional method (9 h). In general, enhancing extraction yield using microwave could be attributed to two main mechanisms, including the rapid temperature increases and molecular friction and rotation. The first one reduces the viscosity of the suspension and increases the cell rupture of raw materials and the second led to the higher ion movement [34]. However, in the present study, the extraction of alginate with the sequential treatment with ultrasound and microwave could not further improve the extraction yield while it slightly decreased the alginate yield to 13.99 %. This could be due to lower temperature (55 ± 2 °C) used in the first step of sequential combination of ultrasound and microwave-assisted method.

### 3.2 FT-IR spectroscopy

**Fig. 3:**
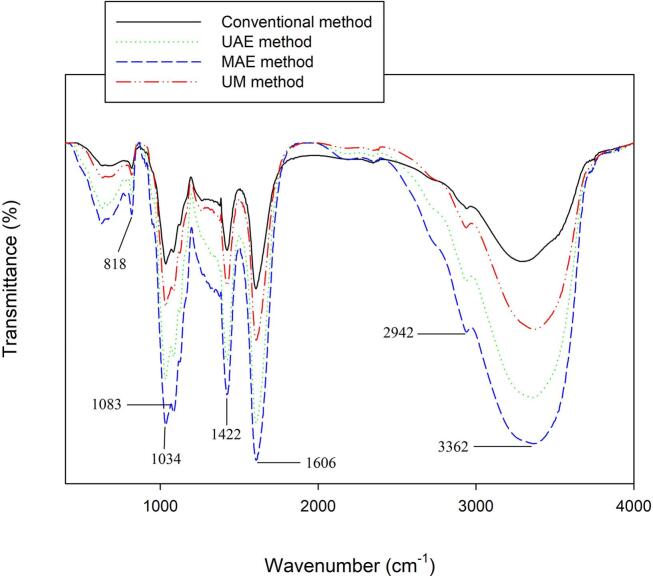
FT-IR spectra of alginates extracted from fucoidan extraction by-products of *N. zanardinii* samples using different extraction methods.

Fig. 3 depicts the FT-IR spectrum of alginate extracted from fucoidan extraction by-products of *N. zanardinii* samples. As can be seen, all the extracted alginates showed similar FT-IR pattern. However, the height and intensity of the characteristic peaks at 1034 cm^−1^, 1422 cm^−1^, 1606 cm^−1^ and 3362 cm^−1^ were increased in the alginate extracted using the green methods especially microwave and ultrasound. The peak at 818 cm^−1^ can be associated with sulphate groups of residues of fucoidan [4]. The peak at 1034 and 1083 cm^−1^ resulted from α-L-guluronic acid and β-mannuronic acid residues, respectively [6]. The peaks at 1422 cm^−1^ and 1606 cm^−1^ attributed to the presence of the COO^−^ symmetric and asymmetric stretching vibration of the carboxylate groups of the mannuronate and guluronate moieties in sodium alginate, respectively [4]. Weak peak around 2942 cm^−1^ was attributed to C—H stretching vibrations. Broad and strong peak at 3362 cm^−1^ corresponding to O—H stretching of hydroxyls groups [35]. Previously, similar FT-IR spectra were reported for alginates of *L. digitata* [10] and *Sargassum ilicifolium* [26] samples. In general, the FT-IR spectrum showed that the application of different extraction methods did not cause any shifts in the characteristic peaks of the extracted alginates and therefore the chemical structure was not substantially changed. Similar results previously reported for the alginate isolated from *S. angustifolium* [6] and *C. peregrina* [8] using different extraction methods including hot water, acidic, alcalase and cellulase enzymes.

### 3.3 Molecular weight of alginate

Molecular weight (*M_w_*) is one of the parameters that affects the different properties and application of polysaccharides, including alginate. This parameter is affected by algal species and their growing conditions as well as extraction techniques. Therefore, various molecular weights of alginate have been reported in the literature. For example, Rhein-Knudsen et al. [4] reported that the average *M_w_* of alginate that was isolated from *S. natans, S. vulgare, P. gymnospora, L. digitata and M. pyrifera*, using a conventional alkaline extraction method, were 569, 514, 482, 756 and 719 kDa, respectively. The average *M_w_* of alginate that was obtained using the ultrasound-assisted technique from *Sargassum muticum* ranged from 80 to 112 kDa [7]. In another study, the average *M_w_* of alginate that was extracted from *S. angustifolium* using hot water, acidic, alcalase and cellulase, were 480, 557, 357 and 356 kDa, respectively [6]. In the present study, the *M_w_* of extracted alginates ranged from 2362 to 3021 kDa at different extraction methods (Fig. 2B). Herein, the alginate isolated using the green methods showed lower molecular weight (2362–3015 kDa) than those extracted using the alkaline method (3021 kDa). Between the green extractions methods, the alginate extracted by ultrasound (2362 kDa) possessed lower *M_w_* than those extracted by the sequential combination of ultrasound-microwave (2608 kDa) and microwave (3015 kDa). This could be considered as favorable property, because according to previous studies [25], the low molecular weight alginate is more bioavailable and absorbable, therefore, its efficacy is higher than the high molecular weight ones. The data obtained in the current study shows the use of ultrasound resulted in the degradation of alginate molecular chains and the formation of short molecular fractions. Sourki et al. [36] reported that the molecular weight of β-D-glucan was reduced by ultrasound compared to that extracted by a conventional method. Degradation of polysaccharide chains including alginate is attributed to the cavitation effect of ultrasound waves and their mechanical energy as well as the act of shear forces formed from rapidly collapsing bubbles. Furthermore, ultrasound could accelerate the collisions between the polymer molecules and solvent molecules, which finally could break the C-C bond of polymer chain [37]. Sonication time and sound amplitude are key factors influencing the reduction of polysaccharide molecular weight through ultrasound treatment [36]. In the current study, it was interestingly found that the microwave treatment was not able to depolymerize and reduce the molecular weight of the extracted alginates. This might be related to the temperature (90 °C) used in this method. Similarly, Alboofetileh et al [22] also reported that the molecular weight of fucoidan extracted from *N. zanardinii* by a microwave method (1184 kDa) was higher than that extracted by hot water (823 kDa), enzymatic (ranging from 634 to 907 kDa), ultrasound (1021 kDa), enzymatic-ultrasound (444 kDa), ultrasound-microwave (748 kDa) and subcritical (670 kDa) methods.

### 3.4 Antioxidant activity

**Fig. 4:**
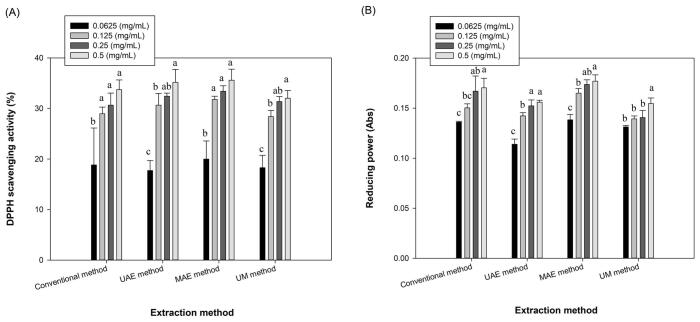
DPPH scavenging (A) and reducing power (B) activities of alginates extracted from fucoidan extraction by-products of *N. zanardinii* samples using different extraction methods (n = 3, means ± SD). The letters a, b, c indicate a significant difference (p < 0.05) between the different concentrations of the alginate in each extraction method.

Reactive oxygen species and free radicals induce different pathological effects including DNA damage, atherosclerosis and carcinogenesis and finally led to the many chronic diseases and aging [38]. There are different antioxidant agents in the living organism to fight with the oxidants. However, in some cases, especially when oxidant levels are exceeded, exogenous antioxidants may also be required. Due to the side effects of synthetic antioxidants [39], natural antioxidants, such as seaweed polysaccharides, have gained significant attention in recent years. Fig. 4A depicts the DPPH scavenging activity of the extracted alginates at concentrations of 0.0625 to 0.50 mg/mL. As can be seen, radical scavenging activity of different extracts was increased with increasing the concentrations of alginate and it ranged from 17.72 to 35.60 %. However, these values were significantly lower than the DPPH scavenging activity of ascorbic acid which was 84.30 % at 100 µg/mL. DPPH scavenging activity of hot water-extracted alginate from *C. peregrina* and *S. angustifolium* were 43 and 39.9 % at 3 mg/mL, respectively [6], [8]. DPPH radical scavenging of alginate from *Cystoseira compressa* and *Cystoseira barbata* were 46 % and 74 % at 0.5 mg/mL, respectively [29], [31]. Purified sodium alginate from *S. vulgare* and its depolymerized fractions prepared using acid hydrolysis (1.5 M HCl) showed a high DPPH radical scavenging activity (64.26 %-92.13 %) at a concentration of 2 mg/mL [35].

Fig. 4B shows the reducing power of extracted alginate from fucoidan extraction by-products of *N. zanardinii* samples. In this assay, a higher absorbance value means stronger reducing power of tested samples. Reducing power of extracted alginates by different extraction methods ranged from 0.114 to 0.177 Abs. As shown, reducing power of the extracted alginates increased with increasing sample concentration and maximum reducing power values were obtained at a concentration of 0.5 mg/mL. The reducing power of ascorbic acid was 0.345 Abs. Reducing powers of alginates from *C. peregrina* and *S. angustifolium* samples were 0.18 and 0.41 abs at 1 mg/mL, respectively [6], [8]. In another study, the reducing power of alginate from *S. muticum* was 0.292 abs at 1 mg/mL [30].

According to the previous studies, the antioxidant activity of alginates originate from their structural features. In this regards, Borazjani et al. [6] reported that the alginate extracted from *S. angustifolium* by enzymatic method, which possess lower molecular weight (356 and 357 kDa), demonstrated higher DPPH radical scavenging and reducing power activities than those extracted by conventional alkaline (480 kDa) or acidic (557 kDa) methods. Similarly, Hifney et al. [13] found that low molecular weight alginate prepared by fungal fermentation or enzymatic pretreatment of the seaweed biomass (*Cystoseira trinodis*) led to enhanced hydroxyl radical scavenging and the ferric reducing antioxidant power of the extracted alginates. However, in the present study, the microwave-extracted alginate which possesses high molecular weight (Fig. 1B) showed the highest DPPH radical scavenging activity. Similarly, a direct relationship was found between molecular weight and reducing power of the extracted alginates. Herein, the alginates extracted by microwave and the conventional alkaline methods with a higher molecular weight showed a higher reducing power than those extracted by the ultrasound and sequential ultrasound-microwave with lower molecular weight. These results showed that the molecular weight of alginates was not the only reason for their antioxidant activity. Supporting this, Hentati et al [31] concluded that the antioxidant properties of polysaccharides are not determined by a single factor but a combination of several related factors. Other factors such as purity, physicochemical properties, M/G ratio, presence of carboxyl and hydroxyl groups of uronic acids also affected the antioxidant activity of alginate [8], [40]. In this regards, previous studies [29], [35] reported that the alginate with a higher proportion of G blocks showed the higher antioxidant activity. Furthermore, other antioxidant compounds such as phenolic compounds which could be co-extracted during the alginates extraction also increase their antioxidant activity [40]. Regarding functional groups, Kelishomi et al. [41] reported that reducing the molecular weight of alginate by thermal treatment increased its antioxidant activity through formation of more functional groups such as carboxyl, carbonyl, and hydroxyl groups and double bonds between C-4 and C-5. Similar to this finding, in the present study, the higher antioxidant activity of alginate extracted by the microwave and ultrasound methods could be attributed to the formation of more carboxylate and hydroxyl groups which are observed in their FT-IR pattern (Fig. 3).

### 3.5 Rheological properties

**Fig. 5:**
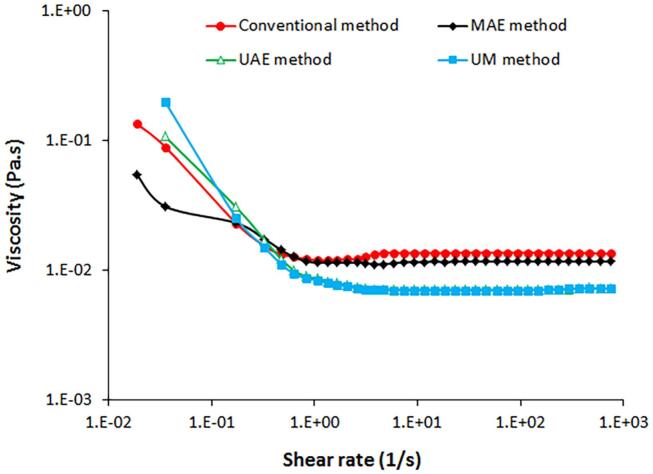
Rheology of alginates extracted from fucoidan extraction by-products of *N. zanardinii* samples using different extraction methods.

One of the important parameters which could affect the application of alginates in different industries is their rheological behavior. In the present study, rheology of alginate solution (1 % w/v) in water was tested by steady-shear flow measurements and the results are shown in Fig. 5. All alginate solutions showed a shear thinning behavior. Similar behavior also reported an aqueous solution of alginate from *Sargassum cristaefolium* and *C. barbata* which showed fluid shear-thinning pseudoplastic [29], [42]. Shear thinning behavior is crucial for industrial applications of alginates because it allows for easy processing and controlled flow under mechanical stress while maintaining viscosity at rest. This property is essential in applications such as food processing, biomedical formulations, coatings, and 3D printing, where alginate solutions must flow smoothly under shear (e.g., during pumping, extrusion, or spraying) but retain their structure once the shear force is removed. Shear thinning also improves stability, uniformity, and efficiency in formulations, reducing energy consumption and preventing clogging in industrial equipment [43].

In addition, the viscosity of the alginate solutions varied depending on the shear rate and the extraction method. Alginates extracted using the conventional and microwave-assisted method showed significantly higher viscosity compared with alginate extracted with the aid of ultrasound and sequential treatment with ultrasound and microwave. As reported, rheological properties of different alginate are dependent on its structural features especially molecular weight [29]. These findings are in line with the lower molecular weight of the alginate extracted using the ultrasound (2362 kDa) and the sequential treatment with ultrasound and microwave (2608 kDa) which showed lower viscosity. Lower molecular weight alginate results in lower viscosity because shorter polymer chains entangle less, reducing resistance to flow. Weaker intermolecular interactions and increased molecular mobility in lower molecular weight alginate further decrease viscosity, as shorter chains have fewer bonding sites and move more freely in solution.

### 3.6 Emulsifying properties

**Fig. 6:**
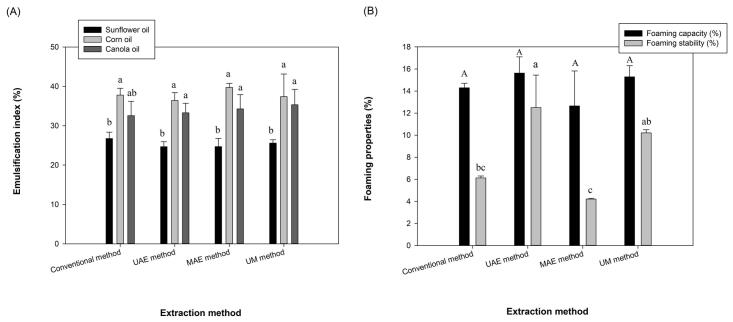
Emulsifying (A) and foaming (B) properties of alginates extracted from fucoidan extraction by-products of *N. zanardinii* samples using different extraction methods (n = 3, means ± SD). The letters a, b, in emulsifying properties indicate a significant difference (p < 0.05) between the different oils in each extraction method. The letters a, b, c indicate a significant difference (p < 0.05) between the foaming stability of the alginate in different extraction methods.

In the current study, sunflower, corn and canola oils were used for the measurement of emulsifying properties of alginate extracted from fucoidan extraction by-products of *N. zanardinii* samples and the results are shown in Fig. 6A. The emulsification index (E24) of the alginates for the tested oils ranged from 24.69 to 41.89 %. In another study, Fawzy et al. [5] reported significantly higher E24 values for alginate of *S. latifolium* samples (57.14–60 %). The E24 of alginate from *C. barbata* for olive, sunflower, corn, soybean, ricin, almond and argan oils were 69.2, 69.2, 75.8, 69.2, 65.8, 69.2 and 69.2 %, respectively [29]. The variation of alginate emulsifying properties could be related to the alginate structure and its solution conditions such as viscosity, temperature, pH and NaCl concentration [5]. In the current investigation, the lowest E24 values of different extracted alginates were measured for sunflower oils. Among the different extracts, the alginate extracted with ultrasound exhibited the lowest E24 across all the studied oils. This could be attributed to its lower molecular weight compared to the other alginates. Results of Liu et al. [44] also demonstrated that the Chinese yam polysaccharides isolated by cellulase enzyme, which possess lower molecular weight (2.2 × 10^6^ g/mol) and apparent viscosity exhibited lower emulsifying activity and stability than those extracted by papain enzyme (2.7–9.5 × 10^6^ g/mol). Protein impurities are another factor affecting the emulsifying properties of polysaccharides. Previous studies [44], [45] have suggested that proteins have a strong tendency to be adsorbed to oil droplets at the oil–water interface and they form a stabilizing layer around the droplets. Brummer et al. [46] also reported that the protein content of polysaccharides positively affected their emulsifying properties and stabilities. In agreement to this, the ultrasound extracted alginate which had a lower protein impurity (5.33 ± 0.12 %), exhibited relatively lower emulsifying properties than the alginates extracted using the conventional, microwave and sequential ultrasound-microwave with protein content of 6.09 ± 0.20 %, 6.29 ± 0.035 and 5.59 ± 0.15 %, respectively.

### 3.7 Foaming properties

Fig. 6B shows the foaming capacity (FC) and foam stability (FS) of different extracted alginates. The FC of alginates (1 % w/v) ranged from 12.66 % to 15.63 % and their FS values also were 4.2–12.5 %. These values are lower than results obtained for water-soluble ulvan polysaccharide from Chaetomorpha linum (FC = 40.12 % and FS = 30.20 %) at a concentration of 1 % [47]. In another study, significantly higher FC (around 73 %) and FS (around 54 %) were reported for ulvan of Ulva intestinalis at a concentration of 3 % [27]. Generally, the foaming properties of alginate are similar to those of other polysaccharides, and are related to their concentration and structural properties. In the current investigation, alginates recovered by the ultrasound and sequential ultrasound-microwave methods possessed higher FC and FS values than those extracted by the conventional and microwave methods. This can be attributed to the molecular weight of these alginates. As can be seen in Fig. 1B, the ultrasound and sequential ultrasound-microwave extracted alginates had lower molecular weight than the other polysaccharides. These data are consistent with the results of Yuan et al. [48] which reported that the ulvan of Ulva prolifera which possesses the lowest molecular weight demonstrated the highest foaming properties (FC = 143 % and FS = 113 %).

## 4 Conclusions

This study investigated the use of green extraction technologies, including ultrasound, microwave, and their combination, for recovering alginate from *N. zanardinii* seaweed by-products following fucoidan extraction. The extraction methods significantly influenced the alginate yield, molecular weight, antioxidant activity (DPPH and FRAP), as well as emulsifying and foaming properties, while FT-IR spectra and rheological properties remained largely unaffected. Among the methods, microwave extraction achieved the highest alginate yield and antioxidant activity, whereas ultrasound and ultrasound/microwave methods produced alginates with reduced molecular weight and enhanced foaming properties. Overall, the findings highlight the potential of green technologies, particularly microwave extraction, as sustainable and efficient alternatives for alginate recovery, contributing to the biorefining of brown seaweed, which is commercially favorable. However, the green extraction process of alginate from fucoidan by-products should be optimized to minimize energy, water, and organic solvent consumption. Further expansion of the biorefinery to recover other value-added products, such as cellulose and glucose, from the alginate extraction residue using green methods could be explored in future studies. These efforts could ultimately enhance sustainability of the industry, maximize seaweed biomass utilization, and support the development of environmentally friendly extraction techniques.

## Data Availability Statement

Data are contained within the article.

## CRediT authorship contribution statement

**Mehdi Alboofetileh:** Writing – review & editing, Writing – original draft, Visualization, Validation, Supervision, Resources, Project administration, Methodology, Investigation, Funding acquisition, Data curation, Conceptualization. **Samira Jeddi:** Writing – original draft, Software, Investigation, Formal analysis. **Mehdi Abdollahi:** Writing – review & editing, Writing – original draft, Visualization, Validation, Supervision, Resources, Project administration, Methodology, Investigation, Funding acquisition, Data curation, Conceptualization.

## Funding

This research received no external funding.

## Declaration of competing interest

The authors declare that they have no known competing financial interests or personal relationships that could have appeared to influence the work reported in this paper.
